# The disproportion of crowd wisdom: The impact of status seeking on Yelp reviews

**DOI:** 10.1371/journal.pone.0252157

**Published:** 2021-06-04

**Authors:** Chao Yu, Drew Margolin

**Affiliations:** Department of Communication, Cornell University, Ithaca, New York, United States of America; Institute for Economic Forecasting, Romanian Academy, ROMANIA

## Abstract

This study shows that while status seeking motivates people to participate in crowdsourcing platforms, it also negatively impacts the bedrock of crowdsourcing–wisdom of crowds. Using Yelp restaurant reviews in 6 cities, we found that motivations of status seeking lead people to review a greater variety of restaurants, and achieving status further encourages this variety seeking as well as the targeting of more expensive restaurants for review. The impact of this individual-level tendency is confirmed by our aggregate-level analysis which shows that restaurants with higher price levels, higher uniqueness levels, and a larger percentage of elite reviews tend to obtain enough reviews to generate wisdom of crowds sooner than other restaurants. This leads to a different kind of distortion to crowd wisdom: an over-representation of status-conferring products and an under-representation of products that are not status-worthy. The findings suggest the importance of studying sources of distortion that are endemic to crowdsourcing itself.

## Introduction

The “wisdom of crowds” is a commonly embraced idea by crowdsourced review sites. The effect of wisdom of crowds refers to the idea that the judgments of many non-experts can be aggregated into a product whose quality rivals or even surpasses that of an expert [1–4]. This provides a theoretical reason to take the content on platforms such as Wikipedia or Yelp seriously.

Crowds, however, are not always wise [5, 6]. Researchers have long recognized this vulnerability in crowdsourcing. For instance, social influences such as herd effect can mislead crowds by homogenizing individual responses, reducing the crowds’ advantage in gathering diverse information [7, 8]. Nonetheless, such social influences may distort the wisdom of any crowd or group, rather than influences that pertain specifically to the dynamics of crowdsourcing platforms. While these general sources of distortion can be a challenge, many can be addressed with policies, such as blinding others’ identifications, without sacrificing important aspects of a crowdsourcing site. Yet, little work has examined sources of distortion that are endemic to crowdsourcing itself.

One such influence is the social incentive for participation in the platform. An important precondition for functional crowd wisdom is large-scale participation. That is, to achieve the effect of the wisdom of crowd, these platforms require that many individuals volunteer their unique knowledge or evaluations. It is also important to note that social incentives are very dependent on the platform and the type of activity [9–13]. For instance, people participate in citizen science project to enhance their reputation [10], and participate in crowdsourcing policymaking to mainly learn about the laws [9].

Status seeking is a type of social incentive for participation that is relevant to crowd wisdom, especially in crowdsourcing platforms that feature opportunities to display or signal status [14]. People seek status to improve their positions in groups or organizations [15]. In particular, Lampel and Bhalla’s study shows that status seeking is a reliable passion that motivates people to continuously participate in online communities [14].

Yet status seeking may also be a source of bias or distortion in what is contributed by the crowd. In particular, like many behaviors governed by social norms, status seeking can homogenize the evaluations and other contributions made by crowd members. For example, since individuals tend to emulate the consumption styles of the higher-class individuals to enhance their own status [16], those observing that high status others have rated a particular product or experience favorably may also rate it favorably.

Status seeking may also have a unique impact on crowdsourcing participation, not only influencing how individuals make evaluations but what they choose to contribute about. In particular, status shows both distinction from inferior groups and identification with superior groups [16, 17]. It is thus partly assigned via association with individuals in these groups as well as the products and experiences typical of them. Status is then obtained by demonstrating knowledge of or familiarity with high status entities—individuals, products, experiences. At the same time, knowledge of or familiarity with low status entities can confer low status, as such knowledge implies either an inability to avoid such entities or an inability to properly discriminate the high from the low. Therefore, people may demonstrate their familiarity with something status-worthy to show their distinction from lower class and association with higher class.

In this light, status incentives on crowdsourcing platforms should encourage participants to over-state their familiarity with high status objects and downplay their familiarity with those of lower status. That is, high status individuals should not only judge low status items as inferior, they should ignore them, leading to a different kind of distortion from the crowd: an over-representation of status-worthy products and an under-representation of lower status products. Thus, the crowd may be wise, but only in regard to status-conferring objects.

Examining the distorting influence of status incentives on crowdsourced behaviors requires analyses at two levels: individual and aggregate. Status seeking is an individual level motive, but the disproportion of crowd wisdom is a result of aggregated individual behaviors that are motivated by status incentives. To carry out analyses at these two levels, we investigate individuals’ restaurant reviews on Yelp, a crowdsourced review site primarily focusing on restaurants. In particular, we investigate Yelp users’ review choices related to two status signals: 1) price levels–an indicator of access to economic resources [16], and 2) display of variety seeking–an indicator of access to cultural resources [18]. We then perform analysis at the aggregate-level (restaurant-level), examining how restaurants that confer those status signals differ with respect to generating wisdom of crowds.

Yelp restaurant reviews provide a unique opportunity for studying these status incentives at both individual- and aggregate-levels. First, status seeking should be an incentive for Yelp users because dining is a common way of displaying status beside fulfilling basic needs [19–21]. Second, Yelp provides a measure of status seeking through its “Elite Squad” program that designates users who post high quality reviews as “elites”. In this study we compare review targets—in terms of the kinds of restaurants chosen for review—between users who seek and eventually achieve elite status (status-achievers) and those who do not achieve the status (non-achievers). This enables us to explore the influence of status seeking motivation on review targets. In addition, we also examine whether gaining the elite status further distinguishes elite users’ review choices from other users. This allows us to investigate the influence of status symbols on review choices. Finally, restaurants’ price levels, categories, and reviews from Yelp users offer an opportunity to test impacts of two status signals on crowd wisdom at an aggregate-level.

Below we briefly define and discuss the motivation of status seeking in crowdsourced review sites as well as the status displays via review targets. Then, we elaborate on how these status displays should differ between status-achievers and non-achievers, and how obtaining elite status should further influence the two status displays. We also test how this individual-level incentive leads to a distortion to wisdom of crowds at an aggregate-level. Methods and data are also described, followed by the results and discussion.

### Status seeking on crowdsourced review sites

Status seeking is a fundamental human motive in social interactions. Status refers to one’s position in a social hierarchy that is determined by prestige, deference or social influence [22, 23]. Studies have shown that people seek status for both external rewards such as economic resources and social relations as well as internal rewards such as psychological and emotional well-being [14, 22, 24].

Status seeking also operates in crowdsourcing platforms and plays an important role in people’s continuous contribution [14]. While some studies show that voluntary contributions are motivated by altruism and reciprocity [25, 26], many studies show that status seeking is the more reliable and sustainable motivation than the others [14, 27, 28]. This dominance of status seeking is partly dependent on features of platforms and types of activities that allow or promote status seeking [10, 14]. Individuals tend to hide their true identities and thus altruism is often not recognized, and reciprocity is hard to guarantee. Status seeking, on the contrary, is not restricted but enhanced by these features. With hidden identities, people are able to selectively present themselves to create more desirable identities that signal status [29]. The ability to broadcast one’s daily life and opinions to a large audience also amplifies the effect of such status display [30, 31]. For instance, individuals can photograph their high-end cuisines, and comment on stays at trendy or expensive hotels in order to enhance their status or reputation.

### Status seeking as a distortion to wisdom of crowds

As status refers to one’s relative standing in a social hierarchy, it works by distinction and association. Individuals seek status to distinct themselves from others [16], and identify themselves with specific groups [17]. That is, they want to be seen as associated with high status groups and disassociated or distant from low status groups. For example, people sometimes pretend to have a British accent because the British English is often associated with higher class [32]. Similarly, familiarity with low status products can downgrade an individual’s status as it can indicate a lack of discernment. For example, the U.S. President George H.W. Bush’s bewilderment with a supermarket scanner was viewed as indicating he was too high status to represent the American people [33]. In other words, high status individuals can not only distinguish the right (high status) things from the wrong (low status) things, but also know about the right things and are ignorant of the wrong things.

Status seeking, therefore, will distort crowd wisdom. Crowds are wise when their contributions and attentions are sufficiently and widely distributed [1, 5]. However, status seekers are not likely to participate regarding the full range of experiences they have had or could have had because they should prefer to be associated with higher class products. For instance, when it comes to restaurant reviews, contributors may want to talk about and display their experience at a trendy new café but downplay or hide their ordinary consumption experiences, such as their experience at Dunkin’ Donuts. Thus, the tendency of focusing on status-worthy products will lead to an over-representation of those products and an under-representation of other products that are not status-worthy.

### Status seeking via two consumption styles

The display of expensive goods and variety seeking are commonly observed by previous scholars as two status signaling consumption behaviors. For decades individuals in higher socio-economic classes could be distinguished by their consumption or possession of upscale products [16, 34]. More recently, scholars also find a tendency for upper-class people to seek a wide variety of products, a phenomenon sometimes described as cultural omnivorousness or variety seeking [18]. These two types of displays are different ways of enabling elites to distinguish themselves from others, but their underlying logic are similar. That is, elites are making these decisions because members of lower classes can’t or won’t follow suit, and those of higher classes will recognize them.

The status signaling via expensive consumption styles is fairly straightforward because it shows direct association with possession of wealth [16]. Economists since Adam Smith [35] have observed that people make purchases not only for the value of the products but also for the status indicated by those products. People’s obsession with expensive products is partly due to their emulation of upper class [16]. Similarly, the consumption of highbrow and lowbrow products can be mapped into higher and lower class [34]. Therefore, status seekers should calculate and choose more expensive products so as to show their association with higher class.

H1: Status seekers tend to visit more expensive restaurants than others.

The display of variety seeking also signals status, but in a different way. The aspiration of variety seeking may appear to be a personal preference, such as sensation seeking or a desire to be healthy [36, 37], but it also has roots in social status distinction that can be achieved by displaying diverse consumption. With the general trend toward supporting diversity and equality, snobbishness became unacceptable in many circumstances because of its overt elitism [18, 38, 39]. In addition, the market economy and mass production have led to the proliferation of cultural products. This has made access to products less distinctive, but at the same time, made knowledge and familiarity with this wide range of products more distinctive, indicating greater sophistication. In this light, variety seeking shows “knowledge and familiarity of as many items as possible and to advance the claim that refinement is to be identified through breadth of experience or awareness” [40 p. 120].

While variety seeking embraces diversity on the surface, it can still be exclusive on a deeper level [38]. Variety seeking signals status by distancing oneself from provinciality, which is a mark of ignorance and lack of education, and thus a sign of lower social class [41]. Therefore, by holding the cultural capital of variety seeking, people signal status by identifying with those from higher socio-economic status, creating strong exclusivity [34, 40, 42, 43]. For example, Jack [44] describes how undergraduate students signal status by discussing exotic vacations that enhance the diversity and exclusivity of their perspectives.

The display of variety seeking is also well-adapted to features of online platforms. By providing a convenient way of knowing different types of products within a certain area, crowdsourced review sites such as Yelp encourage people to seek and display consumption of varied products. In addition, these review sites allow individuals to bypass elite intermediaries–gatekeepers of cultural products such as broadcasters and magazine writers–who once framed and constructed status for particular products [45, 46]. Thus, scarcity of knowledge was reduced. In this context, where diverse experiences are valued and digital information is plentiful, a new scarce commodity emerges–direct experience. That is, those who can access more diverse experiences, and thus displays of such experiences signal wealth and knowledge. In this light, although relatively subtle, the display of diverse consumption experiences should be another means to convey status. Thus,

H2: Status seekers tend to visit a greater variety of restaurants than others.

### The influence of achieving status

The preceding discussion has focused on individuals’ general desire for status. However, there are reasons to expect that achieving status may also impact individual behaviors beyond that induced broadly by seeking. Scholars have shown that the desire for avoiding status loss is stronger than for gaining status even when the benefits of such status is not salient [47–49]. In particular, individuals are willing to put more effort in keeping the status they have than trying to obtain new status. Therefore, the intention to display status should be stronger after obtaining status.

In addition to people’s desire for maintaining status, the extrinsic values of status symbols should also drive people who have obtained status to display status more [50]. Like many offline organizations, crowdsourced review sites create titles (e.g., “local experts” on TripAdvisor, “elite users” on Yelp) that signal status to further encourage contributions from their users. These kinds of titles provide direct distinction from ordinary users and closer association with a small group of users. In online communities, these status symbols also provide users with many other potential benefits such as having their reviews placed on the front page to get more attention from audience. These privileges should further encourage status displays on those platforms. Thus, we expect that,

H3: Compared to before, those who have achieved status tend to visit a) more expensive restaurants, b) a greater variety of restaurants.

### The disproportion of crowd wisdom at an aggregate-level

The preceding discussion on individual-level review choices suggests that restaurants with certain categories should be preferred over others especially among those who have strong motivations for status seeking. In this light, those preferred restaurants are likely to receive a large number of reviews which is the bedrock of wisdom of crowds. Indeed, evaluations of a product often oscillate at the start with a few reviews, stabilizing only as reviews increase. This stable evaluation is the commonly agreed information that reflects the wisdom of the crowd. Thus, if the individual-level review choices tend to favor status-conferring products over others, these products will more easily achieve this “wise”, stable level of reviews.

More precisely, the number of reviews a product receives is time-dependent, so how soon a product can obtain enough reviews to generate wisdom of crowd is particularly important. Theoretically, every product may receive a certain number of reviews if it stays on the market long enough. Yet, slow accumulation of reviews can be problematic because the lifespan of a business may not be long enough to receive enough number of reviews. For instance, the median lifespan of restaurants in US is round 4.5 years, and it is even shorter for small restaurants that have 5 or fewer employees [51]. If restaurants cannot get enough reviews from the crowds within 4.5 years, a large portion of them will go out of business before achieving a wise appraisal.

As people tend to display status via expensive consumption styles and a taste for variety, restaurants with higher price levels and unique categories should achieve wisdom of crowds sooner than others. While the relationship between restaurants’ price levels and expensiveness of consumption styles is straightforward [16], the link between variety taste and restaurant categories is more complicated. A taste for variety is cumulative. That is, to consume a large variety of products, one needs to find out more unique products on the market. For instance, if Italian restaurants are widely available in a place, then status seekers would choose other categories of restaurants if they have already tried Italian cuisines. As status seekers explore more, the number of restaurants that can expand their variety taste become smaller.

Restaurants with a larger percentage of reviews from elite users should also get more reviews. The elite status on Yelp has symbolic value that represents one’s status and authority in the community [52]. In addition, the invitation-only elite users are selected based on their previous contribution to the community, so they act like traditional intermediaries who are supposed to provide high-quality and high credibility reviews. These expectations from elite users thus will attract more reviews from those who want to emulate their consumption experience [16, 17]. Therefore,

H4: Restaurants with a) higher price levels, b) more unique categories, and c) a larger percentage of reviews from elite users, achieve wisdom of crowds sooner than others.

## Materials and methods

### Data

The data for two levels of analyses are sampled from 2019 Yelp academic dataset which is comprised of a complete set of reviews for each business in several cities. For the aggregate-level analysis, we focus on restaurant reviews in the 6 largest cities in the dataset: Las Vegas, Pittsburgh, Phoenix, Charlotte, Madison, and Cleveland. Computing the time needed to achieve wisdom of crowds requires the opening date of each restaurant, but Yelp does not provide such information. We thus use the date of the first review as a proxy for the opening date, so restaurants with only 1 review are excluded in our dataset. The final dataset for the aggregate-level analysis includes 16,627 restaurants with an average of 125.861 reviews (*SD* = 291.087).

Our individual-level analysis is about users’ longitudinal restaurant review choices made in 2017 and 2018 in the 6 cities. Thus, we focus on users whose reviews are mainly posted (50% or more) in one of the 6 cities. Since our hypotheses are about status seeking, we further restrict our sample to users whose behaviors prior to achieving status are observable in the period of 2017 to 2018. Specifically, we make two comparisons at the individual-level: 1) between status seekers and others; 2) within status achievers before and after they achieve their status. The status is operationalized as the elite badge on Yelp which is re-evaluated annually. In our between-user comparison, we focus on review choices in 2017 that are influenced by status seeking motivation but not the elite status (pretreatment). The final dataset for this comparison is comprised of 128,707 reviews on 10,644 restaurants by 63,115 users (367 Yelp elites). To make within-user comparison, we focus on the influence of the elite status (treatment), so we pair status seekers with other users to have better comparability (see details in Analytical Procedures). The final dataset for this comparison contains 16,192 reviews on 5,600 restaurants by 734 users (367 pairs of status-achievers and non-achievers) in 2017 and 2018.

### Measurement

#### Elite status

Yelp introduced its “Elite Squad” program in 2005 to distinguish users who make outstanding contributions to the platform from others. According to Yelp, review quality is one important consideration, but the specific criteria of gaining elite status are not disclosed. Importantly, elite status is not assigned by algorithm but by a dedicated team called the Yelp Elite council. To obtain elite status, users need to submit nomination forms which will be reviewed manually by the council. The elite status is re-evaluated on an annual basis. Thus, in our dataset status seekers were those who did not get elite status in 2017 but achieved their status in 2018.

#### Restaurant price level

Yelp use dollar signs ($) to categorize approximate cost per person for a meal in a restaurant. In particular, “$” means under $10; “$ $” means “$11-$30”; “$ $ $” means “$31-$60”; and “$ $ $ $” means “above $61”. Accordingly, we measured price levels from 1 to 4, and higher level means more expensive.

#### Restaurant uniqueness

Under the category of restaurant, Yelp further categorizes each business by cuisine types. These categories range from detailed menu items, such as “sushi” and “pizza”, to generic cultural categories, such as “Mexican” and “Japanese”. Different from price levels, a restaurant can have multiple categories. A unique restaurant should have uncommon categories on the market such as “Cajun/creole”, or rare combinations of categories such as “Chinese, Italian” fusion restaurants. To measure this uniqueness, we first computed a co-occurrence network of each pair of categories that co-exist in a restaurant. We then built a weighted edge list of all combinations between the categories. Finally, for each restaurant, we calculated the average weight of the possible combinations of its categories. The greater the average weight, the more common the restaurant and thus less unique. We categorize restaurant uniqueness into 3 levels: high (n = 5,532), medium (n = 5,553), low (n = 5,542).

#### Display of variety seeking

We used information entropy [53] to calculate the display of variety seeking. Information entropy captures the even-ness with which different categories appear or are selected. A user with low entropy has only visited one or two categories of restaurants. A user with moderate entropy might have visited many categories of restaurants, but one or two categories much more frequently. Finally, a user with high entropy has visited many categories of restaurants with roughly equal numbers for each type. Our operationalization of the variable is different from the conventional measurement of variety seeking [40, 54] or cultural omnivorousness by volume [20, 55]. Both of these measures use raw counts, essentially measuring how many different categories of cultural products has the individual consumed, with no regard for how much some categories are favored or disfavored compared to others.

We use entropy, rather than these raw-count-based operationalizations, for two reasons. First, we believe entropy is a more fine-grained measure of the construct of interest. If an individual almost always consumes one type of product, but has at one time or another consumed another, this would seem to indicate less variety seeking than when an individual consumes many different products with equal frequency. However, past studies were not able to use this operationalization because they relied on individual recall, which could not give an accurate count of frequency of consumption [20, 56, 57]. With the fine-grained behavioral data available through Yelp, enabling entropy calculations, we believe this measure is an improvement.

Second, we are interested in the display of the behavior to Yelp readers, not the individual’s underlying experience. It is this display that can influence assessment of a reviewer’s eligibility for elite status. This guided our choice of operationalization of the variable. This leads us, first, to use the Yelp list of restaurant types rather than pre-existing cultural categories (as used in cultural omnivorousness studies). These types are what are displayed to Yelp readers. Importantly, this set of categories is quite large (n = 543), and does not come with pre-existing mapping onto cultures. Some categories are clearly identified with a culture (e.g., “Chinese” or “French”), but others would require background knowledge (e.g., “tapas” belongs to Spanish). Thus, it makes more sense for us to treat these categories as is. This is in line with the typical operationalization of variety seeking provided above. Also, because we want to measure the display, what is important is how the reviewer’s choices appear in aggregate to a reader. In this context, a raw count could be very misleading. The overall distribution of reviews is what will create the impression.

For example, a user who has reviewed 497 Japanese restaurants (496 Ramen bar, and 1 Izakaya), but also 1 French restaurant, 1 Chinese restaurant and 1 Thai restaurant would have 4 cultural categories, but it is unlikely that an observer would easily detect this variety (entropy = 0.083). In particular, this user clearly does not display much taste for variety, but would have the same the cultural omnivorousness by volume and the conventional variety seeking as someone who had reviewed 100 of each type. This extreme case can be captured by our entropy measurement (entropy = 2.322) but not the conventional measurement of cultural omnivorousness or variety seeking. Since users’ display of variety seeking changes with their restaurant visits, we calculated the cumulative entropy every time after each of their visits.

#### Time to wisdom of crowds

**Fig 1:**
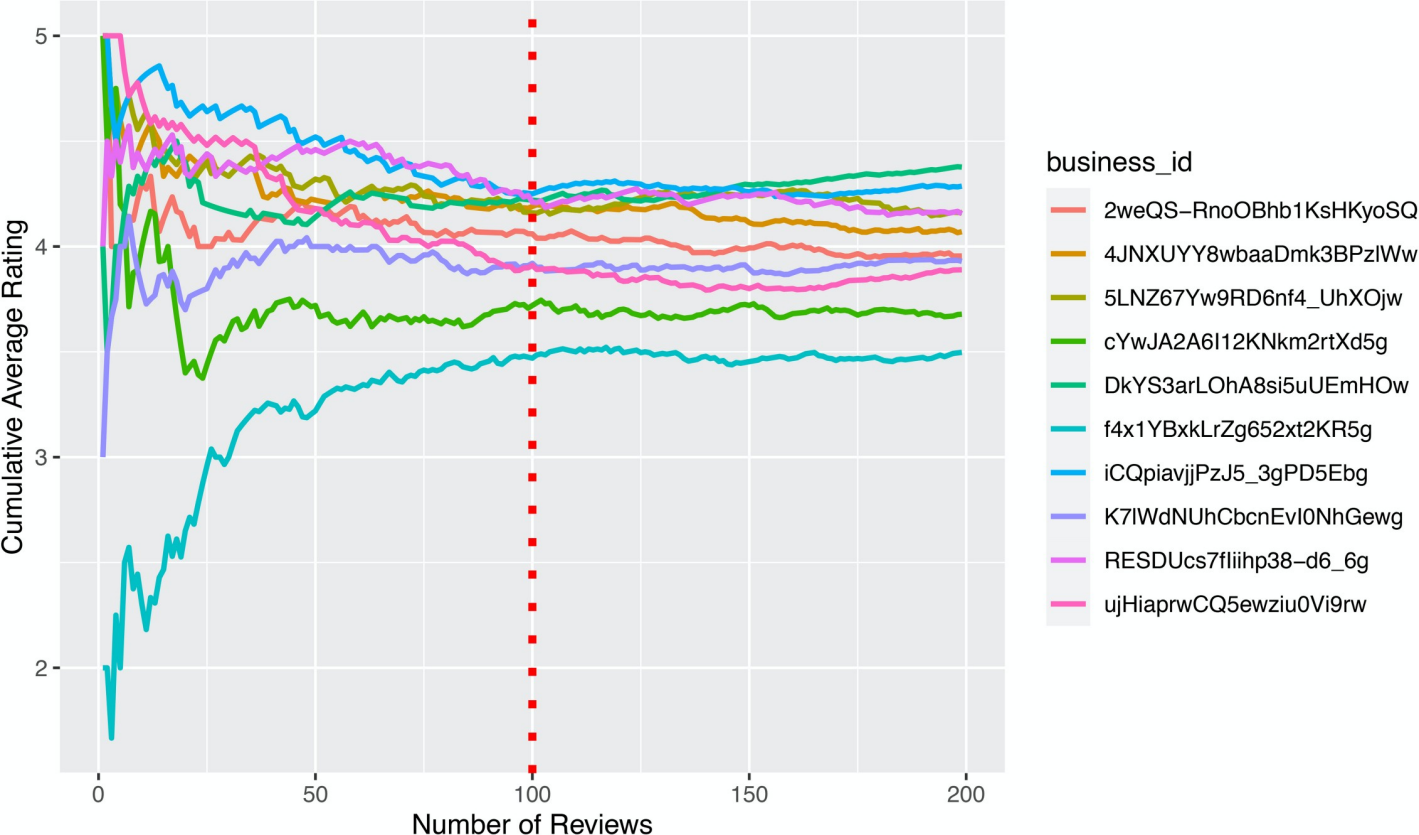
Average ratings stabilize with review volume (first 200 reviews). The cumulative average rating by the number of reviews from the 10 restaurants with most reviews in our dataset. After receiving 100 reviews, the average ratings of the 10 restaurants tend to stabilize.

We define the condition of wisdom of crowds as when a few more future reviews will not dramatically change the overall evaluation of product. Thus, we operationalize this condition by the stabilization threshold in review ratings–the number of reviews needed to have a relatively stable average rating. One important feature on Yelp is that the platform always rounds the average rating to the nearest 0.5. For instance, ratings from 2.75 to 3.25 will all be displayed as 3.0, and ratings from 3.26 to 3.74 will all be displayed as 3.5. In this light, a restaurant achieves wisdom of crowds when a few more future reviews will not change its average rating based on Yelp’s rounding algorithm. We thus first plotted the 10 restaurants with largest number of reviews to see how many reviews the restaurants need so as to have stable average ratings. Based on Fig 1, we found that after receiving 100 reviews, the average rating of the 10 restaurants tend to stabilize. To check the validity of this threshold of 100 reviews, we applied it to the remaining restaurants in our dataset. Because of Yelp’s rounding boundary, we found that after achieving 100 reviews, 93.67% of the restaurants’ ratings will not be affected by the future 10 reviews, 91.84% of them will not be affected by the future 15 reviews, and 90.28% of them will not be affected by the future 20 reviews. Therefore, we use 100 reviews as the stabilization threshold of average ratings, namely a baseline condition in which wisdom of crowds is achieved. The time to wisdom of crowds is thus calculated by the time needed to receive 100 reviews. The measure of time is year.

#### Control variables

At both user- and aggregate-level analyses, we control for their Yelp ages. That is, the length of time that a user’s account and a restaurant’s account had existed on Yelp. As discussed in the preceding paragraphs, one confounding factor that is commonly seen in the wisdom of crowds applications is herd effect [8]. In the case of Yelp platform, people flock to review restaurants that already get attention, i.e. restaurants that receive many reviews on Yelp. To account for this potential confounding factor, we also control for the number of reviews (log transformed) a restaurant had when a user posted his/her reviews.

### Analytical procedures

#### Generalized mixed-effects model

To carry out the between-user comparison, we used a generalized linear mixed-effect model with a binomial distribution. Each user had multiple reviews of businesses in one of the 6 cities. the model included a fixed effect of restaurant price level, display of variety seeking, users’ Yelp age, number of reviews, city, and a random effect of users to predict the achievement of elite status. We used the lmerTest package in R for this analysis.

#### Propensity score matching and difference-in-differences

To get credible causal inference of achieving elite status on individuals’ review choices, we should control for other individual-level differences. Similar to social incentives being specific to platforms, users who eventually achieved elite status might be very different from others regardless of the elite status. For instance, some users are enthusiastic participants in the community, but others rarely contribute to the platform. Therefore, even without achieving the elite status, the former would be quite different from the latter. To control for these differences, we match each status achievers with a comparable user who was very likely qualified for the elite status but “randomly” did not obtain it. More specifically, we match users based on a propensity score constructed from the following 7 variables in 2017 (before anyone achieved elite status): average price level of the restaurant visited, display of variety seeking, average rating, number of reviews, number of three votes (useful, cool and funny) on users’ reviews. These variables are all directly related to users’ performance on Yelp, and thus should capture the user-level differences that may influence the chance of achieving elite status. An R package, MatchIt, was used for the propensity score matching. The matching procedure yields 367 Yelp elites (treatment) and 367 matched non-elites (control). After matching, both groups of users are indistinguishable in terms of the 7 variables (see Table 1). We then used a quasi-experimental design–difference-in-differences method–to test the effect of achieving elite status on people’s review choices. We used the lfe package in R for this analysis.

**Table 1:** Results of *t* Test on the variables that could capture users’ qualifications for elite status before and after propensity score matching.

	Before Matching				After Matching			
	Status-Achievers	Non-Achievers	*t*(df)	*p*	Status-Achievers	Non-Achievers	*t*(df)	*p*
	*M (SD)*	*M (SD)*			*M (SD)*	*M (SD)*		
Price levels	1.315 (0.811)	1.722 (0.585)	9.589 (368.23)	< .001	1.315 (0.811)	1.345 (0.818)	0.498 (731.95)	.619
Variety seeking	4.265 (2.641)	2.641 (0.862)	-40.31 (371.40)	< .001	4.265 (2.641)	4.274 (0.711)	0.178 (727.55)	.858
Average rating	4.103 (0.512)	3.657 (1.527)	-16.265 (405.11)	< .001	4.103 (0.512)	4.057 (0.776)	-0.954 (633.61)	.340
Review count	9.114 (8.841)	2.013 (2.522)	-15.384 (366.35)	< .001	9.114 (8.841)	9.177 (11.618)	0.082 (683.42)	.934
Cool votes	3.635 (8.473)	0.336 (2.088)	-7.459 (366.26)	< .001	3.635 (8.473)	3.371 (15.688)	-0.284 (562.77)	.776
Funny votes	0.236 (0.524)	0.129 (0.468)	-3.906 (369.43)	< .001	0.236 (0.524)	0.267 (1.022)	0.512 (545.60)	.609
Useful votes	0.769 (1.022)	0.428 (0.974)	-6.377 (369.90)	< .001	0.769 (1.022)	0.798 (2.190)	0.227 (518.24)	.821

#### Cox proportional hazards regression analysis

In our dataset for aggregate-level analysis, 11,578 out of 16,627 restaurants were *censored*, i.e., they did not get to the event of wisdom of crowds (receiving 100 reviews). Censored observations should be included in our analysis because those restaurants might need longer time to have the event. We thus used the Cox hazard model that takes the censored observations into account. Specifically, we modeled the relationship between the hypothesized restaurant features and the time to the event. We also included a separate fixed intercept within each city to account for the heterogeneity between cities. An R package, *survival*, was used for the analysis.

## Results

Our first two hypotheses postulate that status seekers are likely to visit more expensive restaurants (H1) and visit a greater variety of restaurants (H2) than others. The mixed-effect model in Table 2 shows no significant relationship between people’s average price level of their restaurants visits and their probability of achieving elite status (*B* = -0.133, *SE* = 0.210, *p* = .525). This does not support H1. However, the model reveals that those who seek a greater variety of restaurants are more likely to achieve status (*B* = 1.104, *SE* = 0.134, *p* < .001). This supports H2.

**Table 2:** Generalized mixed-effects model of variables predicting status seekers.

	Dependent variable: Become Elite
Price level	-0.133
	(0.210)
Display of variety seeking	1.104***
	(0.134)
Users’ Yelp age	-0.074
	(0.063)
Review count	-0.004
	(0.108)
Constant	138.659
	(127.542)
Observations	128,707
Log Likelihood	-2,515.573
Akaike Inf. Crit.	5,053.146
Bayesian Inf. Crit.	5,160.564

*Note*: *p<0.05; **p<0.01

***p<0.001.

**Fig 2:**
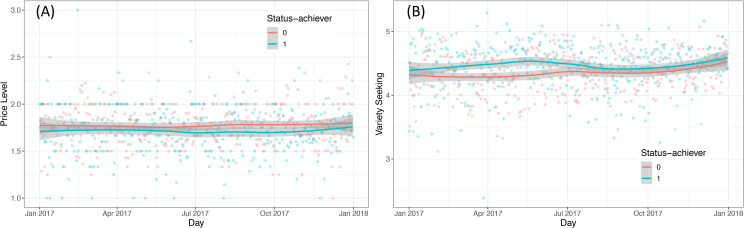
Pre-treatment trends of average price level and variety seeking between status-achievers and non-achievers. The daily average price level (A) and the display of variety seeking (B) between status-achievers and non-achievers before achieving statue in 2017 (during the pretreatment period). There is no obvious difference in the trends of two restaurant choices in the pretreatment period as is displayed above. This supports the parallel pretreatment trend assumption.

We used a difference-in-differences approach to test our third hypothesis which predict that compared to before, gaining status leads people to visit more expensive restaurants (H3a), and a greater variety of restaurants (H3b). One key assumption of difference-in-differences approach is the parallel trend in pretreatment. To test this assumption, we calculated and plotted the average of two restaurant choices everyday separately for status-achievers and non-achievers in the pretreatment period (see Fig 2). Visual inspection on the plots shows no obvious difference in the trends of two restaurant choices between the two groups of users. This supports the parallel pretreatment trend assumption. Table 3 presents the summary of the difference-in-differences results with robust standard errors clustered at the user level to account for serial correlation [58]. Model 1 and 2 show that achieving status leads people to choose more expensive restaurants (*B* = 0.051, *SE* = 0.026, *p* = .025), and a greater variety of restaurants (*B* = 0.114, *SE* = 0.029, *p* < .001). Therefore, H3 is supported.

**Table 3:** Difference-in-differences estimates of achieving status on price level and display of variety seeking.

	Dependent variables:	
	Price Level	Display of Variety Seeking
	(1)	(2)
Diff-in-Diff	0.051*	0.114***
	(0.026)	(0.029)
Observations	15,825	16,192
R^2^	0.170	0.370
Adjusted R^2^	0.089	0.310
Residual Std. Error	0.540 (df = 14410)	0.675 (df = 14775)

*Note*. Robust *SE*s are parenthesized (robust against clustering by users).

*p<0.05; **p<0.01

***p<0.001. There are 367 reviews made toward restaurants that do not display price levels on their Yelp page.

**Fig 3:**
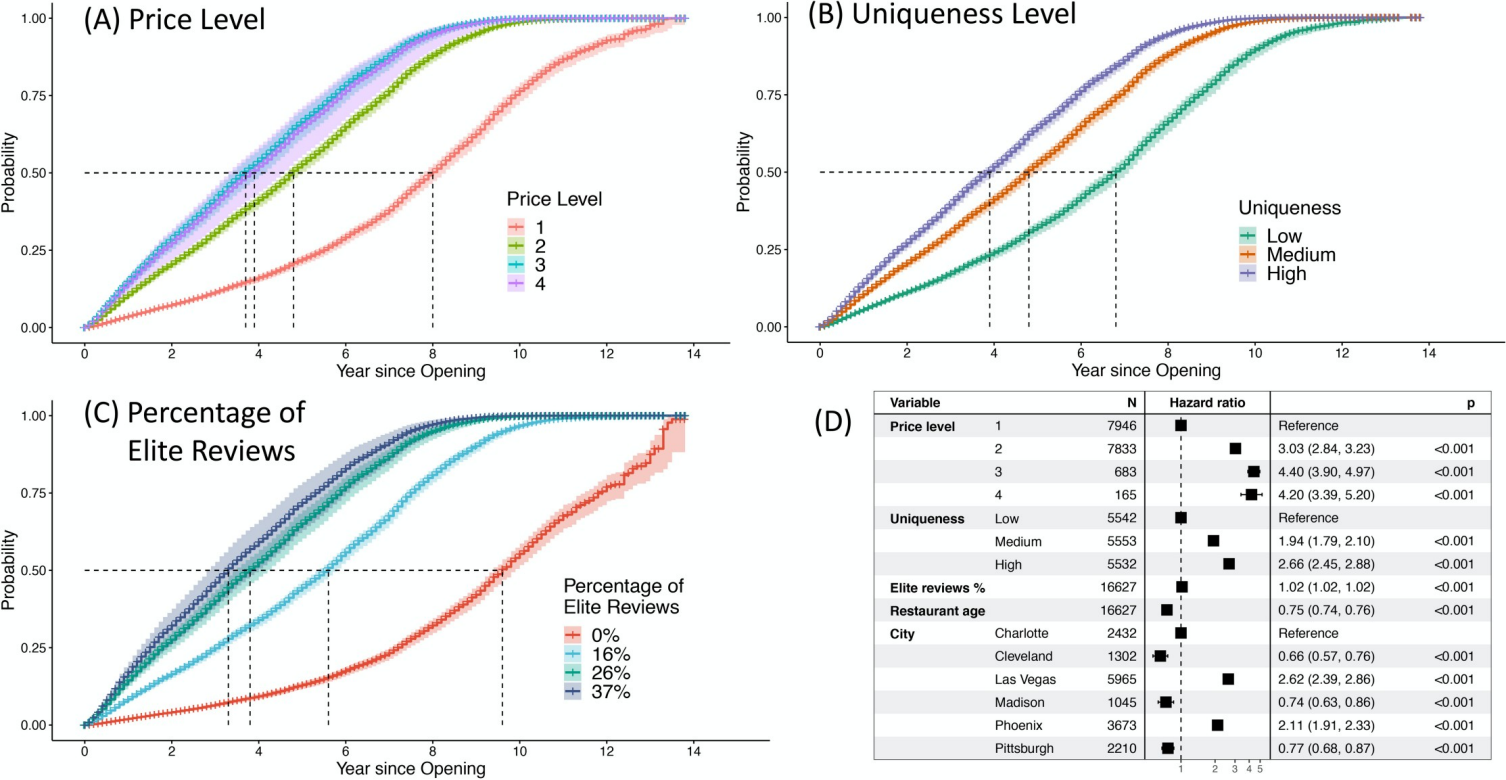
Incidence of achieving crowd wisdom. Cumulative incidence of achieving crowd wisdom (CW) with different price levels (A), uniqueness levels (B) and percentage of elite reviews (C) (zeroth to third quartile) at average restaurant age and in Las Vegas. The dotted lines show the time needed for 50% of restaurants in each category to achieve CW. The table (D) shows the hazard ratio (ratio of getting CW) among different categories of restaurants.

Per H4, restaurants with a) higher price levels, b) more unique categories, and c) a larger percentage of reviews by elite users, achieve wisdom of crowds sooner than others. As showed in Table 4, the Cox proportional hazards model reveals that compared with 1-dollar-sign restaurants, 2-dollar-sign restaurants (*B* = 1.108, *SE* = 0.033, *p* < .001), 3-dollar-sign restaurants (*B* = 1.482, *SE* = 0.062, *p* < .001), and 4-dollar-sign restaurants (*B* = 1.435, *SE* = 0.109, *p* < .001) have a higher incidence (hazard) of achieving wisdom of crowds. This also means that in terms of the ratio of achieving wisdom of crowds (hazard ratio), 2-, 3-, and 4-dollar-sign restaurants are 3.028, 4.403, 4.198 times higher than 1-dollar-sign restaurants, respectively. The model also shows that compared with restaurants of low uniqueness, those of medium uniqueness (*B* = 0.661, *SE* = 0.042, *p* < .001), and high uniqueness (*B* = 0.978, *SE* = 0.041, *p* < .001) have higher incidence (hazard) of achieving wisdom of crowds. In terms of the ratio of achieving wisdom of crowds (hazard ratio), medium- and high-uniqueness restaurants are 1.938, and 2.659 times higher than low-uniqueness restaurants, respectively. Finally, the percentage of elite reviews also positively influence the incidence, (*B* = 0.021, *SE* = 0.001, *p* < .001). Therefore, H4 is supported. See Fig 3 for a visual illustration.

**Table 4:** Results of cox proportional hazards model predicting the incidence of achieving crowd wisdom.

	Dependent variable: Achieving Crowd Wisdom		
	Coef.	SE	Hazard Ratio
Price level 2 ($ $)	1.108***	0.033	3.028
Price level 3 ($ $ $)	1.482***	0.062	4.403
Price level 4 ($ $ $ $)	1.435***	0.109	4.198
Uniqueness-medium	0.661***	0.042	1.938
Uniqueness-high	0.978***	0.041	2.659
Elite reviews %	0.021***	0.001	1.021
Restaurants’ Yelp age	-0.286***	0.006	0.751
Observations	16,627		
R^2^	0.286		
Max. Possible R^2^	0.996		
Log Likelihood	-42,147.440		
Wald Test	5,266.460*** (df = 12)		
LR Test	5,593.954*** (df = 12)		
Score (Logrank) Test	5,729.109*** (df = 12)		

*Note*. *p<0.05; **p<0.01

***p<0.001.

Fixed intercepts for cities are included.

## Discussion

This study explores the influences of status seeking on wisdom of crowds at both individual- and aggregate-levels. Using Yelp restaurant reviews, we found that, at the individual-level, the motivation of status seeking leads people to review a greater variety of restaurants, and achieving status further encourages this display of variety seeking as well as the targeting of more expensive restaurants for review. These findings show that status seekers tend to review products that confer status, especially among those who have achieved status. Our aggregate-level analysis confirms this tendency by showing restaurants with higher price levels, higher uniqueness levels, and larger percentage of elite reviews tend to achieve wisdom of crowds sooner than other counterparts. This leads to an over-representation of status-conferring products and an under-representation of products that are not status-worthy.

Our study links the literature of motivations behind crowdsourcing with the literature of impacts on people’s participation in crowdsourcing. Current studies on crowdsourcing often focus on either individual-level behaviors [14] or aggregate-level phenomenon [6]. Either level of analyses is important to better understand the functioning of crowdsourcing but linking the two levels together provides a more wholistic picture of this phenomenon. As our findings indicate, a positive motivation at individual level may lead to negative effects when aggregated. This demonstrates the need for more conversations between the two literatures.

Our study also provides several implications. First, crowdsourced review sites that feature cultural consumption, such as platforms like Spotify, IMDb, and Yelp, should be mindful about the side effects of encouraging users’ participation by status incentives. These dynamics would also apply to the reviews of cultural products, such as books or movies, within more general sites such as Amazon, even if they do not influence the reviews of more purely functional items (e.g., windshield wipers). For cultural products, although the motivation of status seeking will lead to more contributions from users, this motivation may also distort the wisdom of crowd which is a foundation for crowdsourcing platforms. While keeping users’ participation is important, balancing the representation of their contributions is also vital for them to provide an overall benefit to their users.

Second, products on review sites should be conscious of the time they need to achieve enough reviews so as to generate wisdom of crowds, especially for products that are not status-conferring. Our findings show that cheap, common restaurants need significantly more time than those expensive, unique restaurants in terms of achieving wisdom of crowds. In particular, it takes about 8 years for 50% of the 1-dollar-sign restaurants on Yelp to receive 100 reviews (see Fig 3). Yet, the median lifespan of restaurants in US is no more than 5 years [51], so most low-end restaurants may not achieve wisdom of crowds before they go out of business. A previous study shows that reviews from elite users are almost twice as impactful as other reviews on restaurants’ revenues [59]. Our study reveals that reviews from elite users will also help restaurants attract more reviews. This indicates the importance of having local experts’ and celebrity users’ attentions for a business to survive and thrive.

Finally, our study brings up the discussion on wisdom of crowds in terms of its fundament: having enough contributions from crowds. There has been extensive research on different aspects of wisdom of crowds such as the influence of peer pressure, the herding effects [6, 8], and the quality and credibility of crowd wisdom [2, 60]. These studies are based on the assumption of already having enough contributions from crowds. However, before we can discuss these influences and features of wisdom of crowds, it is important to know that many objects may not even have enough attention from the crowds to generate wisdom. This disproportion of crowd wisdom has been recognized in previous research [2] but it deserves more attention as it may exist in many other crowdsourcing platforms.

We also find an unexpected result in our study: status seekers do not visit more expensive restaurants than others (as H1 is not supported). There are several reasons that may explain this result. First, as we have discussed earlier, overtly showing off expensive consumption styles may have become unpopular in many circumstances because of the influence of inclusionary ideology [38, 39]. Thus, status seekers may downplay their preference for expensive restaurants. Second, restaurants on Yelp are categorized into only 4 price levels which is a rough estimation of cost per meal with a small variation. For instance, the price difference between a cheap restaurant and a Michelin 3-star restaurant could be hundreds of dollars, but such difference is only 3 dollar-signs on Yelp. Third, even if two individuals choose the same restaurant, their actual bills may differ a lot. These possibilities may also explain why H3a is supported but with a p-value (0.025) close to the cutoff of 0.05.

### Limitations and future directions

Our study comes with a few limitations. In between-user analysis, we distinguished status seekers from other users by checking whether they achieved status in the future (2018). This was a rough estimation since those who had strong motivations of status seeking but did not achieve the status in 2018 should also be considered as status seekers but were not identified as so. Future study may attempt to use better measures such as review language styles to identify their motivations.

Secondly, as we have described, the display of variety seeking can also be influenced by the inclusionary ideology [38]. This more internally motivated explanation is difficult to tease apart from external influences in our observational data. Future research should explicitly explore the influence of such different, internal motivation. In particular, the influence of inclusionary ideology could be experimentally tested or explored through surveys or interviews.

Finally, our current finding about status seeking being a motivation that may distort the effect of wisdom of crowds is only limited to specific crowdsourcing platforms. As we have emphasized in preceding paragraphs, motivation factors are very platform- and activity-specific, so we think our findings can only be applied to crowdsourcing platforms that promote or provide opportunities to display status. However, we are interested in the motivation of status seeking in other crowdsourcing platforms/activities, and we think this is the next step for future studies.

## Conclusions

This study focuses on the impacts of status seeking on wisdom of crowds at both individual- and aggregate-levels. Using Yelp restaurant reviews, we found that the seeking and maintenance of status play an important role in users’ participation in the platform: users who are motivated to obtain and maintain status gravitate toward status-worthy products, which will lead to a disproportion of crowd wisdom. We thus call for attention to sources of distortion that are endemic to crowdsourcing itself, because such distortion is fundamental to the concept of crowdsourcing.
